# Close-Range 3D Hyperspectral Measurement System with a Physics-Guided Spectral Correction Model

**DOI:** 10.3390/s26113396

**Published:** 2026-05-27

**Authors:** Zhiyuan Liu, Wenxiu Wan, Ziru Yu, Zhiqie Jiang, Xiangyang Yu, Youliang Zhang, Shengkang Luo, Yuchen Guo, Ke Chen

**Affiliations:** 1 State Key Laboratory of Optoelectronic Materials and Technologies, School of Physics, Sun Yat-Sen University, Guangzhou 510275, China; liuzhy229@mail2.sysu.edu.cn (Z.L.); wanwx3@mail2.sysu.edu.cn (W.W.); jiangzhq6@mail2.sysu.edu.cn (Z.J.); cesyxy@mail.sysu.edu.cn (X.Y.); chenk35@mail.sysu.edu.cn (K.C.); 2 School of Electronics and Communication Engineering, Sun Yat-Sen University, Shenzhen 518107, China; 3 Guangzhou Guangxin Technology Co. Ltd., Guangzhou 510300, China; guoyuchen@guangxin.tech; 4 Department of Plastic and Reconstructive Surgery, The Affiliated Guangdong Second Provincial General Hospital of Jinan University, Guangzhou 510220, China; almazyl@hotmail.com (Y.Z.); luoshk@gd2h.org.cn (S.L.)

**Keywords:** hyperspectral imaging, 3D reconstruction, spectral correction

## Abstract

**Highlights:**

**What are the main findings?**

- A close-range 3D hyperspectral measurement framework was developed by integrating a structured-light 3D module with a hyperspectral imaging module, achieving sub-40-μm sphere-fitting RMS residuals and an effective spectral resolution of 7 nm.
- A physics-guided spectral correction model (3D-LFSC) was proposed for geometrically complex surfaces, improving spectral consistency by more than 10% compared with existing correction methods.

**What are the implications of the main findings?**

- The proposed framework improves the reliability of reflectance-related spectral measurements on geometrically complex surfaces, where conventional white-board calibration and Lambertian-based correction are often inadequate.
- The corrected 3D hyperspectral point clouds support downstream optical sensing tasks, such as color consistency analysis on facial surfaces, and show potential for close-range spectral assessment of biological samples.

**Abstract:**

Three-dimensional (3D) hyperspectral point clouds provide both surface geometry and spectral information, offering a promising tool for close-range surface characterization. However, reliable reflectance-related spectral measurement on complex surfaces remains challenging because camera-recorded spectral signals are strongly affected by non-uniform illumination, surface geometry, and the spectral response of the imaging system, while existing correction methods are often limited by Lambertian assumptions and narrow spectral capacity. In this work, we present a close-range 3D hyperspectral measurement framework with geometry-aware spectral correction that integrates a structured-light 3D measurement module with a hyperspectral imaging module. The system enables the acquisition of fused 3D hyperspectral data with a sphere-fitting RMS residual below 40 μm and a spectral resolution of 7 nm. To improve spectral correction on geometrically complex surfaces, we propose a physics-guided spectral correction model, termed 3D light-field spectral correction (3D-LFSC), which is inspired by the geometric dependence described by the bidirectional reflectance distribution function (BRDF) and uses measurable geometric information to model geometry-dependent spectral variation. Because the system adopts a cross-polarized illumination–detection configuration, the corrected spectra should be interpreted as diffuse-dominant apparent reflectance estimates under the fixed system configuration, rather than complete surface reflectance. Experiments on surfaces with different geometries and reflectance properties show that the proposed method improves spectral consistency by more than 10% compared with existing methods. The framework also demonstrates applicability to chromaticity-related analysis on facial surfaces, indicating its potential for close-range spectral measurement of complex biological surfaces.

## 1. Introduction

Hyperspectral imaging (HSI) represents a significant advancement in optical sensing technology, capturing spatially resolved spectral data across hundreds of contiguous wavelength channels. This technology can extract spectral information from objects and enable rapid, non-destructive analysis of their chemical composition. Compared with multispectral imaging, which typically provides only tens of wavelength bands, HSI can generate two-dimensional (2D) images with hundreds of contiguous spectral bands [1]. With such high spectral resolution, HSI enables molecular identification with transformative applications across remote sensing [2,3,4,5], biomedicine [6,7,8,9], agriculture [10,11,12,13], and food quality assessment [14,15,16,17]. While conventional HSI generates hyperspectral images, it lacks depth information, limiting 3D surface analysis. Conversely, 3D reconstruction techniques, such as time-of-flight imaging [18], stereo vision [19], laser triangulation [20], and structured light [21], captures geometric features but lack spectral information. By combining 3D reconstruction with HSI, the complementary advantages of the two modalities can overcome these limitations, enabling the simultaneous acquisition of HSI data and 3D point clouds to generate 3D hyperspectral point clouds.

Existing 3D hyperspectral imaging systems can be broadly divided into three groups: registration-based systems, non-registration-based systems and deep-learning-based systems. The most significant advantage of non-registration-based systems is their ability to directly output 3D hyperspectral point clouds without extra registration algorithms. Representative works by Luo et al. employ optical cage architectures to establish point-to-point correspondence between hyperspectral and 3D imaging modules, achieving high depth accuracy and spectral resolution [22,23]. However, these systems strongly rely on complex opto-mechanical components, resulting in large system volume, high cost, and limited flexibility in practical applications. Another strategy is to acquire 3D point clouds and HSI data on a single sensor [24,25,26,27,28]. However, the imaging speed [25,28], spectral resolution or depth accuracy [24,26,27] are limited.

In contrast, registration-based systems acquire HSI data and 3D point clouds using separate modules and subsequently fuse them through calibration and registration. Therefore, registration-based systems can avoid complex optical paths and hardware designs, allowing greater flexibility and scalability. However, precise system calibration and registration are vital, and misalignment errors can severely degrade the quality of the fused 3D hyperspectral point clouds. These works focus on registration-based systems [29,30,31].

Regarding the deep-learning-based systems, related work focuses on reconstructing 3D point cloud from existing HSI [32] or reconstructing HSI from RGB-D data [33]. Although these methods reduce hardware complexity, their performance strongly relies on training data, and they struggle to simultaneously achieve high depth accuracy and high spectral resolution in 3D hyperspectral point clouds.

Despite these advances in system design and data fusion, reliable reflectance-related spectral measurement on complex surfaces remains a major challenge in close-range 3D hyperspectral imaging. In close-range hyperspectral imaging, the recorded spectral signal is affected by spatial illumination non-uniformity [34] and distance-dependent attenuation, surface-normal variation, local incidence and observation geometry, and angular effects [35]. As a result, even a homogeneous material may produce different recorded spectra at different surface positions and orientations. The conventional white-board calibration uses a reference plate with stable reflectivity in the working spectral range, which is placed at the same position as the sample to minimize illumination non-uniformity. Although this method is effective for flat samples, it performs poorly for spectral normalization of samples with complex surface shapes. Variations in surface normals and interreflection among complex surface structures alter the reflected radiance reaching the hyperspectral camera.

To mitigate these effects, researchers have proposed spectral correction and reference-based normalization methods. Recent developments have focused on establishing a geometric-spectral model: including 3D white-reference geometric-spectral libraries for low-tilt surfaces [36], and a data-driven neural network correction trained from polytetrafluoroethylene (PTFE) standards’ 3D hyperspectral point clouds achieving 25 wavelength bands [37,38]. Although these works can employ 3D information to improve spectral consistency, current methodologies cannot simultaneously optimize depth accuracy, spectral resolution, and spectral correction bandwidth. Furthermore, data-driven correction models are generally constrained by Lambertian assumptions, narrow spectral capacity and lack physical interpretability, which restricts their applicability to non-Lambertian surfaces.

Current systems still confront three fundamental limitations: inherent complexity-accuracy trade-offs in optical design, Lambertian model constraints in spectral correction, and limited spectral channel capacity for spectral correction. This paper presents a 3D hyperspectral measurement framework with geometry-aware spectral correction that combines a structured-light 3D measurement system with a hyperspectral imaging system, enabling sequential acquisition of 3D point clouds and hyperspectral images. The main novelty of this work lies in a physics-guided spectral correction model, named 3D light-field spectral correction (3D-LFSC), guided by the BRDF description of geometry-dependent reflection. Here, “light-field” does not refer to light-field-camera imaging, but to the geometry-dependent illumination–observation field under the fixed measurement configuration. It accounts for illumination effects and geometry-spectral interactions beyond Lambertian models. In experiments on surfaces with different geometries and reflectance properties, the effective spectral channel capacity for 3D-LFSC increases 3.7-fold, delivering a spectral consistency improvement of over 10% compared to state-of-the-art methods. The proposed method aims to improve the consistency and repeatability of apparent reflectance estimates on complex surfaces.

## 2. System Design and Methods

### 2.1. Optical Design of the System

**Figure 1:**
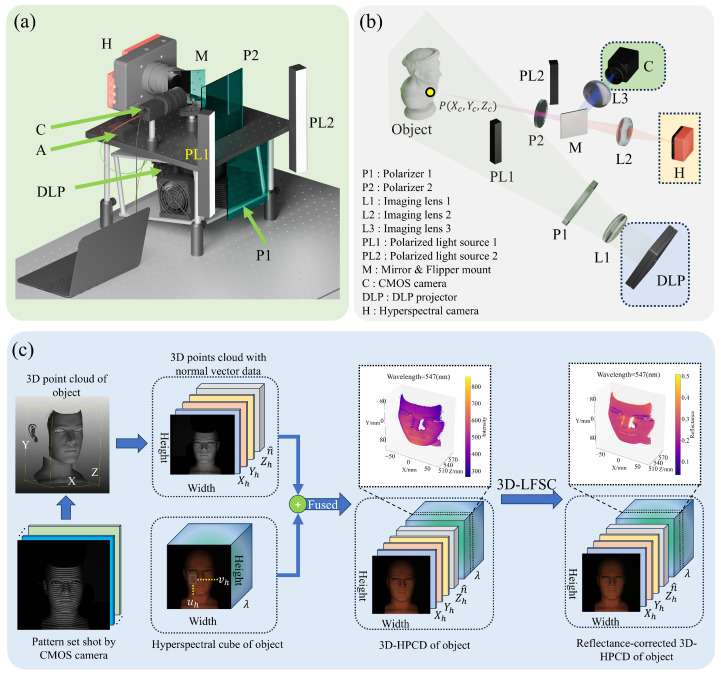
The framework of our work. (**a**) Schematic diagram of 3D hyperspectral measurement framework. H: Hyperspectral camera; C: CMOS camera; A: Amplifier; DLP: DLP projector; PL1: Polarized LED light source 1; PL2: Polarized LED light source 2; P1: Polarizer 1; P2: Polarizer 2; M: Mirror and flipper mount. (**b**) Optical path of 3D hyperspectral measurement framework. (**c**) Data processing workflow.

The 3D hyperspectral measurement framework (Figure 1a) has three modules: (1) A fringe projection profilometry (FPP)-structured light system consisting of a CMOS camera (MV-CS050-10GC, Hikvision, Hangzhou, China; 2448 × 2048 pixels) and a digital light processing (DLP) projector [M26EGLD (LC), AnHua Optoelectronics, Shenzhen, China; 1920 × 1080 pixels] generates high-precision 3D point clouds. An amplifier circuit ensures precise projector–camera synchronization. (2) A hyperspectral imaging system (SpecimIQ camera, Specim, Oulu, Finland; 512 × 512 pixels, native spectral range of 400–1000 nm with 204 channels) acquires HSI data. In the current system configuration and data analysis, the effective operating spectral range is 430–700 nm with 92 usable channels, corresponding to an effective spectral resolution of 7 nm (full width at half maximum, FWHM). (3) Two polarized strip light sources (CST-2POB32738-W, CST Automation, Dongguan, China; 430–700 nm) provide linear polarized illumination for HSI acquisition. The projector (tilted upward at approximately 10°) and the CMOS camera are mounted on the lower and upper optical plates, respectively. A flipper-mount mirror is used to enable optical path switching. The whole system is on an optical vibration isolation platform.

Figure 1b illustrates the optical principle of 3D hyperspectral measurement framework. Both light sources (polarized strip light source and DLP projector) and imaging devices (hyperspectral camera and CMOS camera) are equipped with polarizers. The polarization axes of light sources are aligned to each other and are orthogonal to the imaging devices. This optical design can suppress specular reflection during the acquisition of 3D point clouds and HSI. Therefore, the recorded hyperspectral signal mainly represents the cross-polarized, diffuse-dominant component of the reflected light, rather than the complete reflected signal from the surface.

During 3D point-cloud acquisition, the flipper mount is in the flipped-up position, and the CMOS camera is operational. The DLP projector projects a set of coded patterns onto the target, and the CMOS camera captures each image of the object encoded by the projected patterns. Through image decoding and 3D reconstruction, 3D point clouds of the object are generated, providing the object’s 3D coordinates $P(X_{c},Y_{c},Z_{c})$. During HSI acquisition, the flipper mount is in the flipped-down position, and the hyperspectral camera is operational. The two polarized strip light sources provide polarized illumination for HSI acquisition, allowing the collection of spectral information $I_{P}(\lambda_{1},\lambda_{2},\ldots )$ of the object. The co-axial design minimizes parallax between different imaging sensors, enhancing the accuracy of 3D hyperspectral point cloud fusion during subsequent computational processing.

The total acquisition time of the current system is mainly determined by the hyperspectral imaging module. Under the current camera settings, hyperspectral acquisition takes approximately 60 s, while the structured-light 3D acquisition requires only a few seconds. Therefore, the total acquisition time for one 3D hyperspectral measurement is approximately one minute.

After data acquisition through the 3D hyperspectral measurement framework system, a set of images captured by the CMOS camera and HSI data acquired by the hyperspectral camera are obtained. The images from the CMOS camera serve as the raw input for 3D reconstruction and are subsequently processed into 3D point clouds. As shown in Figure 1c, the CMOS camera captures a sequence of images in which coding patterns are projected onto the object surface. These images are then converted into a 3D point cloud through 3D reconstruction algorithms, denoted as $P(X_{c},Y_{c},Z_{c})$.

Prior to registering the 3D point cloud with the HSI data, we estimate the normal vectors of the point cloud—a crucial step for subsequent spectral correction. Upon completing this step, a 3D point cloud enriched with normal vector information is obtained, represented as $P(X_{c},Y_{c},Z_{c},n_{X},n_{Y},n_{Z})$. The HSI data are then registered with the 3D point cloud, establishing a one-to-one correspondence between the 3D coordinates of each point and its corresponding hyperspectral pixel values. Based on this correspondence, the 3D point cloud and HSI data are fused to generate the raw 3D hyperspectral point clouds, expressed as $P(X_{c},Y_{c},Z_{c},n_{X},n_{Y},n_{Z},I_{P})$. Furthermore, we construct a 3D-LFSC model, a geometry-aware neural network guided by radiometric considerations of surface reflection, to process the raw 3D hyperspectral point clouds and derive spectrally corrected 3D hyperspectral point clouds with geometry-normalized apparent reflectance estimates.

### 2.2. Generation of 3D Point Clouds

A 3D point cloud is obtained by a FPP structured-light system. The projector sequentially illuminates the target surface with three encoded pattern sets: (1) sinusoidal fringe patterns; (2) 8-bit Gray code patterns; and (3) complementary 8-bit Gray code patterns. Surface geometry optically modulates these projected patterns, inducing deformations observed by a camera positioned at a triangulation angle. Depth information becomes encoded within both the fringe phase and Gray code orders. The intensity of phase-shifted fringe patterns captured by the camera follows:

(1) $$I_{m}=A\left(u_{c},v_{c}\right)+B\left(u_{c},v_{c}\right)cos\left[\varphi \left(u_{c},v_{c}\right)+\frac{2m\pi }{4}\right]$$

where $I_{m}$ denotes the intensity of the $m^{th}$ phase-shifted pattern (m=0,1,2,3); $A\left(u_{c},v_{c}\right)$ represent camera pixel coordinates’ background illumination; $B\left(u_{c},v_{c}\right)$ denotes camera pixel coordinates’ fringe modulation amplitude; and $\varphi \left(u_{c},v_{c}\right)$ denotes camera pixel coordinates’ phase distribution. The wrapped phase map is obtained through phase demodulation [39]:

(2) $$\varphi \left(u_{c},v_{c}\right)=\tan^{-1}\left[\frac{\sum_{m=0}^{3}I_{m}\sin\left(\frac{2m\pi }{4}\right)}{\sum_{m=0}^{3}I_{m}\cos\left(\frac{2m\pi }{4}\right)}\right]$$

The resulting wrapped phase $\varphi \left(u_{c},v_{c}\right)$ exhibits periodicity within $\left[-\pi ,\pi \right]$ and cannot be uniquely mapped to projector pixels. To resolve the fringe order for absolute phase determination, complementary Gray code patterns are binarized. The first 7 Gray code images are decoded to obtain the primary fringe order $k_{1}\left(u_{c},v_{c}\right)$, while all 8 patterns yield the complete order $k\left(u_{c},v_{c}\right)$. These orders are then combined to derive the secondary fringe order $k_{2}\left(u_{c},v_{c}\right)$ [40]:

(3) $$\left\{\begin{array}{c} k_{2}\left(u_{c},v_{c}\right)=k_{1}\left(u_{c},v_{c}\right)+1,\frac{k\left(u_{c},v_{c}\right)}{2}>k_{1}\left(u_{c},v_{c}\right)\\ k_{2}\left(u_{c},v_{c}\right)=k_{1}\left(u_{c},v_{c}\right),\frac{k\left(u_{c},v_{c}\right)}{2}=k_{1}\left(u_{c},v_{c}\right) \end{array}\right.$$

The secondary fringe order $k_{2}\left(u_{c},v_{c}\right)$ compensates for decoding errors caused by misalignment between the primary order $k_{1}\left(u_{c},v_{c}\right)$ and wrapped phase $\varphi \left(u_{c},v_{c}\right)$. Absolute phase $\Phi \left(u_{c},v_{c}\right)$ is then reconstructed through phase-domain segmentation [40]:

(4) $$\Phi \left(u_{c},v_{c}\right)=\left\{\begin{array}{c} \varphi \left(u_{c},v_{c}\right)+2\pi k_{2}\left(u_{c},v_{c}\right),\varphi \left(u_{c},v_{c}\right)\leq -\frac{\pi }{2}\\ \varphi \left(u_{c},v_{c}\right)+2\pi k_{1}\left(u_{c},v_{c}\right),-\frac{\pi }{2}<\varphi \left(u_{c},v_{c}\right)\leq \frac{\pi }{2}\\ \varphi \left(u_{c},v_{c}\right)+2\pi k_{2}\left(u_{c},v_{c}\right),\varphi \left(u_{c},v_{c}\right)>\frac{\pi }{2} \end{array}\right.$$

where $\Phi \left(u_{c},v_{c}\right)$ denotes the absolute phase distribution. This enables calculation of the projector’s vertical coordinate mapping:

(5) $$v_{p}\left(u_{c},v_{c}\right)=\frac{\Phi \left(u_{c},v_{c}\right)}{2\pi }v_{0}$$

with $v_{0}$ being the projector’s vertical pixel resolution. The 3D world coordinates $\left(X_{W},Y_{W},Z_{W}\right)$ are subsequently recovered through perspective projection [41]:

(6) $$\left[\begin{array}{c} X_{W}\\ Y_{W}\\ Z_{W} \end{array}\right]={\left[\begin{array}{ccc} h_{c11}-h_{c31}u_{c} & h_{c12}-h_{c32}u_{c} & h_{c21}-h_{c33}u_{c}\\ h_{c21}-h_{c31}v_{c} & h_{c22}-h_{c32}v_{c} & h_{c23}-h_{c33}v_{c}\\ h_{p21}-h_{p31}v_{p} & h_{p22}-h_{p32}v_{p} & h_{p23}-h_{p33}v_{p} \end{array}\right]}^{-1}\left[\begin{array}{c} h_{c34}u_{c}-h_{c14}\\ h_{c34}v_{c}-h_{c24}\\ h_{p34}v_{p}-h_{p24} \end{array}\right]$$

The transformation matrix H incorporates intrinsic and extrinsic parameters, which are derived from camera and projector calibration parameters.

### 2.3. Generation of 3D Hyperspectral Point Clouds

Figure 1c shows the generation of 3D hyperspectral point clouds with normal vectors. Surface normal vectors are first estimated by defining localized neighborhoods constrained to a maximum of 16 nearest neighbors within a 1 mm radius—parameters optimized to mitigate normal vector errors at surface discontinuities. Spatial registration is then achieved through coordinate transformation between the industrial camera frame ${(X}_{c},Y_{c},Z_{c})$ and hyperspectral camera frame ${(X}_{H},Y_{H},Z_{H})$:

(7) $$\left[\begin{array}{c} X_{h}\\ Y_{h}\\ Z_{h} \end{array}\right]=R_{HC}\left[\begin{array}{c} X_{c}\\ Y_{c}\\ Z_{c} \end{array}\right]+T_{HC}$$

where $R_{HC}$ and $T_{HC}$ denote the rotation matrix and translation vector obtained via Zhang’s method [42]. Then, hyperspectral pixel coordinates are transformed through perspective projection:

(8) $$\left[\begin{array}{c} u_{h}\\ v_{h}\\ 1 \end{array}\right]=\frac{1}{Z_{h}}K_{h}\left[\begin{array}{c} X_{h}\\ Y_{h}\\ Z_{h} \end{array}\right]$$

where $K_{h}$ is the intrinsic parameter matrix of hyperspectral camera, and $\left(u_{h},v_{h}\right)$ is the coordinates of hyperspectral camera. Combining Formulas (7) and (8) with the distortion correction, we can achieve pixel-level fusion of the 3D point cloud and HSI.

### 2.4. Limitations of Explicit BRDF-Based Reflectance Modeling

For most objects, the reflected light consists of specular and diffuse components, which cannot be adequately described by the Lambertian reflection law alone. Therefore, BRDF formulations, such as the Cook–Torrance model, are useful for illustrating how reflected radiance depends on diffuse or specular components and illumination–observation geometry. The Cook–Torrance BRDF expresses the reflected radiance as a weighted combination of diffuse and specular components [43,44,45]:

(9) $$f_{r}=k_{d}f_{d}+k_{s}f_{s}$$

where $k_{d}$ and $k_{s}$ denote the weighting coefficients of the diffuse and specular reflection components, respectively; $f_{d}$ and $f_{s}$ denote the corresponding diffuse and specular BRDF terms. In this work, the Cook–Torrance formulation is not used to estimate material-specific BRDF parameters. Instead, it serves as a radiometric motivation to indicate that the camera-recorded spectral signal is jointly affected by surface geometry, illumination, viewing direction, and material-dependent reflection components.

**Figure 2:**
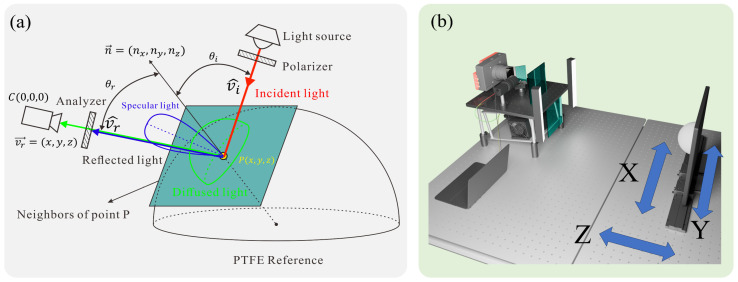
Physical motivation of the proposed 3D-LFSC model and acquisition of training data. (**a**) Radiometric motivation based on geometry-dependent reflection described by the Cook–Torrance BRDF formulation. (**b**) Data acquisition of training data.

To minimize the influence of specular components, we use a pair of orthogonally oriented polarizers in both the illumination and imaging paths (Figure 2a). This design can effectively suppress specular components, which allows the observed reflective light to be dominated by diffuse reflection. It should be noted that, under this cross-polarized configuration, the measured spectral signal does not correspond to the complete surface reflectance. Instead, it represents a diffuse-dominant, cross-polarized reflectance-related signal under the fixed illumination–observation configuration. Under this configuration, the reflected radiance received by the camera can be expressed as [45]:

(10) $$L_{r}\left(\hat{v_{r}};\lambda \right)=\int_{\Omega }L_{i}\left(\hat{v_{i}};\lambda \right)\max\left(0,cos\theta_{i}\right)k_{d}\left(\lambda \right)f_{d}\left(\lambda \right)d\Omega_{i}$$

where $L_{r}\left(\hat{v_{r}};\lambda \right)$ represents the reflected radiance along the observation direction; $L_{i}\left(\hat{v_{i}};\lambda \right)$ represents the incident radiance from the illumination direction; $\theta_{i}$ represents the angle between surface normal vector and the incident direction. The integral over the hemisphere Ω accounts for all incident paths satisfying $cos\theta_{i}>0$, with $\theta_{i}$ denoting the incidence angle relative to the normal surface. The wavelength-dependent parameters $k_{d}\left(\lambda \right)$ and $f_{d}\left(\lambda \right)$ are determined by material optical properties. Although the polarization setting simplifies the problem through minimizing specular components, the remaining diffuse components still depend on the incident illumination and surface geometry.

In a camera-centered coordinate system, a structured-light system can directly measure surface point 3D coordinates (x,y,z) and their corresponding normal vectors $\left(n_{x},n_{y},n_{z}\right)$. Consequently, the viewing direction vector can be accurately and directly obtained. However, as illustrated in Equation (10), accurate estimation of reflectance-related quantities still depends on explicit knowledge of the incident light directions and illumination geometry, such as the vector from the light source point to each surface point and the corresponding incidence angle. In practical 3D hyperspectral imaging systems, the illumination geometry is often complex and difficult to characterize because of unknown light source positions and spatially varying illumination distributions. As a result, these illumination-related parameters cannot be reliably measured or estimated. Therefore, directly applying explicit BRDF-based radiometric correction methods, even under simplified conditions, is infeasible for practical 3D hyperspectral imaging systems. This limitation motivates the proposed 3D-LFSC: instead of explicitly solving the BRDF parameters or illumination geometry, the model uses measurable 3D coordinates and surface normals to implicitly learn a geometry-dependent reference spectrum that accounts for the combined influence of illumination and surface geometry.

In this work, the BRDF formulation is not used as an explicit forward model for fitting material-specific BRDF parameters. Instead, it is used to identify the radiometric dependency of the recorded spectral signal on illumination, viewing geometry, and surface orientation. This dependency motivates the use of measurable 3D coordinates and surface normals as model inputs. The PTFE hemisphere is not used to train a Cook–Torrance BRDF model, but to provide a geometry-varying reference response of the measurement system under the fixed illumination–observation configuration. The proposed 3D-LFSC then learns this geometry-dependent reference response and uses it for spectral normalization.

### 2.5. 3D-LFSC Model

As discussed in the previous section, direct BRDF-based radiometric modeling is difficult in the proposed system because the illumination geometry and material-specific BRDF parameters are not explicitly known. In contrast, 3D coordinates and surface normal vectors can be reliably acquired by the structured-light module.

This motivates a reformulation of the spectral correction problem. Rather than explicitly estimating BRDF parameters or illumination geometry, 3D-LFSC uses measurable geometric features to implicitly model their combined influence on the camera-recorded spectral signal. Under the conditions of orthogonally oriented polarizers, the specular component is effectively suppressed, and the observed spectra are dominated by the diffuse component. In this case, each surface point’s measured spectrum can be separated into a wavelength-independent amplitude term and a wavelength-dependent spectral shape term. The amplitude term primarily modulates the overall energy level of the spectrum, while the spectral shape encodes the relative distribution of energy across wavelengths.

As Equation (10) illustrated, the amplitude of the diffuse reflected radiance depends on surface geometry, such as surface normal orientation, viewing direction, and distance-related attenuation, which collectively determine the effective contribution of incident illumination to the observed radiance. Although the explicit illumination directions are unknown, their influence is implicitly embedded in the geometric configuration of surface points. These factors jointly determine the amount of incident illumination contributing to the observed radiance. Therefore, we assume that the spectral amplitude can be modeled as a function of measurable geometric attributes, without explicitly estimating the incident illumination directions or spatial light distribution.

In contrast, the spectral shape term is determined by the spectral distribution of the illumination source and the intrinsic reflectance properties of the material. Therefore, directly modeling the absolute reflectance spectrum of arbitrary objects under unknown illumination is impractical. We reformulate the problem as the estimation of a geometry-dependent reference spectrum, which characterizes the combined effects of illumination and geometry. Therefore, the neural network does not output BRDF parameters, material reflectance parameters, or illumination directions. Instead, the predicted reference spectrum represents the expected camera-recorded response of a spectrally flat reference target at the same geometric configuration and is then used to normalize the measured spectrum of the target object. The geometry-normalized apparent reflectance is estimated as:

(11) $${\hat{R}}_{app}(\lambda )=\frac{I_{object}\left(\lambda \right)}{I_{object}^{ref}\left(\lambda \right)}$$

where $I_{object}(\lambda )$ represents the raw spectral signal of the object after standard dark-field subtraction recorded by the hyperspectral camera, and $I_{object}^{ref}(\lambda )$ denotes the geometry-dependent reference spectral signal predicted by the proposed model. The output ${\hat{R}}_{app}(\lambda )$ is interpreted as a geometry-normalized, diffuse-dominant apparent reflectance under the cross-polarized and fixed illumination–observation configuration of the proposed system, rather than an absolute surface reflectance, a complete surface reflectance, or a full bidirectional reflectance quantity.

An ideal reference target should exhibit spectrally flat and stable reflectance, such that its observed spectral shape is dominated by the illumination spectrum rather than material-dependent variations. Therefore, in the training setup, a PTFE hemisphere is used as a geometry-varying spectral reference due to its near Lambertian behavior with a reflectivity of approximately 95% across the 400–1500 nm range [46,47]. In addition, the PTFE hemisphere provides a continuous and diverse distribution of surface normal vectors, enabling the model to observe a wide range of geometric configurations under fixed illumination conditions. This design facilitates effective learning of geometry-dependent amplitude modulation while maintaining a consistent spectral shape, which is essential for decoupling geometric effects from spectral characteristics in the proposed framework.

Although we decompose the reference spectrum into shape and amplitude, the real-world imaging system inevitably deviates from the ideal model. In practice, residual specular components due to polarizer leakage, multiple scattering, and sensor nonlinearity may lead to spectral shape distortions. These effects typically manifest as smooth, correlated variations across wavelengths, suggesting that they can be effectively represented in a low-dimensional subspace. Accordingly, we introduce a spectral residual term modeled using principal component analysis (PCA), which captures low-dimensional deviations from the dominant spectral shape. The PCA basis is computed from the residual spectra of the reference measurements after removing the primary shape component. To preserve the physical interpretability of the amplitude–shape decomposition, the residual component is constrained to be orthogonal to the primary spectral shape. This constraint ensures that the residual term only accounts for shape variations, while the overall spectral energy is solely controlled by the amplitude term.

Based on these assumptions, the proposed 3D-LFSC model predicts the spectral amplitude and low-dimensional residual coefficients from geometric features, and reconstructs the reference spectrum by combining the dominant spectral shape with the learned residual correction.

**Figure 3:**
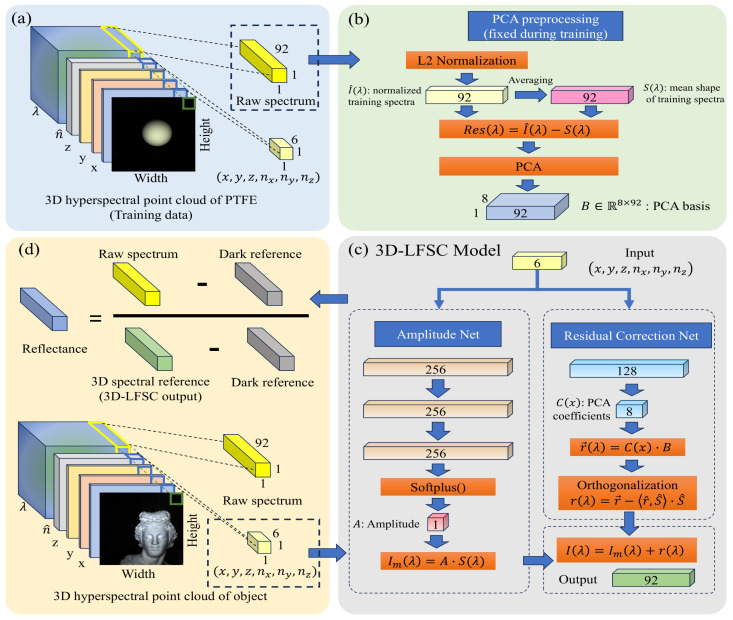
Overall workflow and architecture of the proposed 3D-LFSC. (**a**) Fused 3D hyperspectral point clouds of PTFE hemisphere used as training data; (**b**) pipeline of training data preprocessing; (**c**) architecture of the 3D-LFSC model, which predicts geometry-dependent spectral amplitude and residual coefficients from 3D coordinates and surface normals to reconstruct a reference spectrum; (**d**) application of the trained model for spectral correction, where the predicted reference spectrum is used to normalize the measured spectral signals and estimate geometry-normalized apparent reflectance.

As shown in Figure 3a, the training data for the 3D-LFSC model consist of fused 3D hyperspectral data of a PTFE hemisphere. Each point of training data is associated with its spatial coordinates, surface normal vector, and measured spectrum. The training data acquisition methods are shown in Figure 2b.

Figure 3b illustrates the spectral representation adopted in this work. Each measured spectrum is decomposed into a dominant spectral shape component and a residual component. The dominant shape is defined as the average spectrum of the training set, justified by the fact that, under orthogonal polarization and using a spectrally flat PTFE reference, the observed spectral variations across surface points are primarily dominated by geometry-dependent amplitude modulation rather than wavelength-dependent shape changes. This assumption is further supported by the high spectral consistency of the training data, with cosine similarity exceeding 0.985. The residual component captures low-dimensional deviations from this dominant shape and is represented using PCA, yielding a compact set of residual coefficients. As a result, each spectrum is parameterized by a scalar amplitude term and a low-dimensional vector of PCA coefficients.

Figure 3c illustrates the functional pipeline of the proposed 3D-LFSC model. The model takes geometric attributes of each surface point as input and predicts two geometry-dependent quantities: a spectral amplitude term and a set of low-dimensional residual coefficients. The amplitude term represents the geometry-induced modulation of spectral energy, while the residual coefficients account for deviations from the dominant spectral shape in a compact subspace. These predicted quantities are combined with the predefined spectral basis to reconstruct the reference spectrum for each surface point.

Figure 3d illustrates the application of the trained 3D-LFSC model. For a target object, the model predicts a reference spectrum for each surface point based solely on its geometric attributes. The geometry-normalized apparent reflectance is then estimated by normalizing the measured spectral signal using the predicted reference spectrum according to Equation (11). The output is a spectrally corrected 3D hyperspectral point cloud, in which spectral measurements are decoupled from geometric and illumination-induced variations.

## 3. Results

### 3.1. System Characterization

The intrinsic and extrinsic parameters, along with the distortion coefficients of the CMOS camera, hyperspectral camera, and DLP projector, are calibrated using Zhang’s method [42]. Specifically, the CMOS camera and hyperspectral camera are used to capture images of a checkerboard calibration target (comprising 11 × 9 corners with a spacing of 20 mm) placed at multiple orientations. These images are then processed with Zhang’s calibration algorithm to determine the camera parameters.

Once the CMOS camera is calibrated, the DLP projector is calibrated indirectly using the CMOS camera as a reference. Since the DLP projector is not an imaging device, it cannot be calibrated directly with Zhang’s method. Instead, a pair of complementary checkerboard patterns are projected onto the calibration target, and the corresponding images are captured by the CMOS camera to extract the projected corner points. The image coordinates of these corners in the projector frame are predefined, while their corresponding world coordinates are obtained from the calibrated checkerboard geometry. With both the projector image coordinates and world coordinates of the corners established, Zhang’s method is applied to compute the intrinsic and extrinsic parameters of the DLP projector. Supplementary Table S1 summarizes the calibration parameters of the 3D hyperspectral measurement framework.

**Figure 4:**
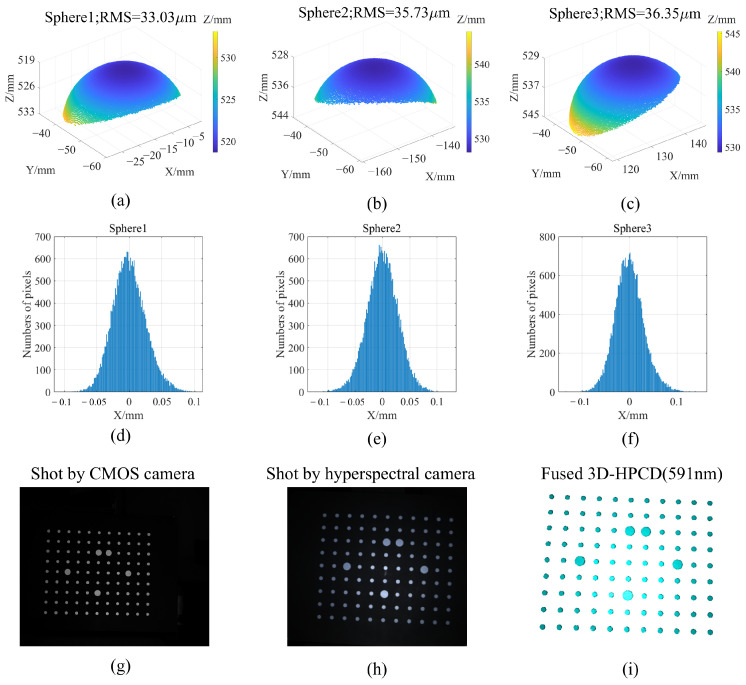
Evaluation of 3D reconstruction and hyperspectral-point cloud fusion. (**a**–**c**) Three representative reconstructed point clouds of the certified ceramic sphere. (**d**–**f**) Corresponding spatial distributions of point-to-sphere residuals after sphere fitting. The sphere-fitting RMS residual was used to characterize local surface reconstruction precision and depth repeatability. (**g**) CMOS camera image of the spot calibration board. (**h**) Hyperspectral camera image of the calibration board. (**i**) Fused 3D hyperspectral point cloud at 591 nm. Hyperspectral spots and reconstructed 3D points show consistent spatial correspondence.

The 3D reconstruction performance was evaluated using a certified ceramic sphere (Ø30.0075 ± 0.005 mm) [48,49]. For each reconstructed point cloud, a sphere was fitted to the measured 3D points. The sphere-fitting root-mean-square (RMS) residual was used to characterize local surface reconstruction precision and depth repeatability, while the radius error, defined as the absolute difference between the fitted radius and the certified radius, was used as a complementary indicator of dimensional accuracy. Three representative reconstruction results and their corresponding point-to-sphere residual distributions are shown in Figure 4a–f. In addition, nine repeated measurements were conducted at different lateral positions on the same measurement plane, and the detailed results are summarized in Supplementary Table S2.

Hyperspectral-point cloud fusion was validated using a spot calibration target (Figure 4g–i). The CMOS image (2448 × 2048 px) and hyperspectral image [512 × 512 px, Band 65 (591 nm)] show precise spatial registration in the fused 3D hyperspectral point clouds. Quantitative alignment accuracy was confirmed through: (1) Visual correspondence between spectral spots with 3D points; (2) reprojection error of 0.45 px calculated via Zhang’s calibration. Table 1 illustrates the performance of the 3D hyperspectral measurement framework.

Table 2 illustrates the comparison between our 3D hyperspectral measurement framework and representative 3D multispectral or hyperspectral imaging systems. In contrast to the binocular multispectral imaging (BMSI) system of Wan et al. [50], our system achieves a higher spectral resolution of 7 nm versus 20 nm, enabling the discrimination of finer spectral features. In 3D reconstruction, our system employs active structured-light projection and achieves a mean sphere-fitting RMS residual of 36.39 μm at a working distance of 530 mm. The BMSI system, relying on binocular stereo vision, provides a depth accuracy of 0.89 mm at 1036 mm and is critically dependent on surface texture. Consequently, for weakly textured or feature-similar objects, our system provides improved reconstruction accuracy under the tested close-range condition. Relative to the 4D multi-frame co-encoded spectral imaging system proposed by Qi et al. [24], our system reports a mean sphere-fitting RMS residual of 36.39 μm, compared with the reported 3D reconstruction accuracy of 0.1 mm in that work. Moreover, that work did not address geometric distortions on complex surfaces in spectral correction. When compared to the system of Gevaux et al. [51], several differences can also be observed. Our system reports a mean sphere-fitting RMS residual of 36.39 μm, whereas Gevaux’s system reports a depth error of approximately 0.4 mm, representing an improvement of more than one order of magnitude. In spectral resolution, our system provides an effective resolution of 7 nm with 92 usable channels, while Gevaux’s system uses a liquid-crystal tunable filter with a bandwidth of 10 nm and only 31 channels. The higher spectral resolution and denser wavelength sampling allow our system to resolve more subtle spectral features, facilitating refined analysis of biological parameters such as skin pigmentation and blood oxygen saturation. Unlike the portable 4D snapshot hyperspectral imager of Luo et al. [27], our system reports a mean sphere-fitting RMS residual of 36.39 μm, compared with the reported depth error of 55.7 μm in Luo’s system. In spectral resolution, our system achieves 7 nm with 92 usable channels, compared to 10 nm of Luo’s system, enabling finer spectral discrimination. Regarding spectral correction, Luo’s system only employs white-board normalization to remove source spectral influences and does not address geometry-induced spectral distortions. In contrast, our system introduces the BRDF-motivated 3D-LFSC model, which uses measurable 3D coordinates and surface normals to reduce geometry- and illumination-induced spectral variations on geometrically complex surfaces, leading to an improvement in spectral consistency of more than 10%.

### 3.2. Model Performance

To comprehensively evaluate the proposed spectral correction method, we conduct experiments on two representative samples with distinct surface geometries and reflectance-related properties: a silicone face sample and a small-radius plastic sphere. The silicone face represents a practical scenario with moderate curvature variation and complex surface properties, while the plastic sphere serves as an extreme test case where strong curvature induces pronounced illumination variation and intensity fall-off, resulting in highly spatially varying reflectance.

We compare our method (3D-LFSC) against two baselines: neural reference field (NeREF) [38] and white-board calibration (a conventional reference-based method). Because the original NeREF model was developed for lower-band multispectral data, a direct application to our 92-band hyperspectral setting requires dimensional adaptation. To construct a comparison under matched spectral dimensionality, we retained the original network depth and layer connectivity of NeREF and scaled the hidden-layer widths approximately in proportion to the output dimensionality (92/25 ≈ 3.68), while leaving the overall design as unchanged as possible. Both NeREF and 3D-LFSC were trained on the same training data and evaluated using the same preprocessing pipeline and performance metrics. Therefore, the adapted NeREF serves here as a bandwidth-matched baseline for spectral correction in our hyperspectral setting.

To quantitatively assess spectral consistency across spatial locations, we adopt the coefficient of variation (CV) as the primary evaluation metric. For a given wavelength λ, CV is defined as the ratio of the standard deviation to the mean of the corrected apparent reflectance values across spatial samples:

(12) $$CV\left(\lambda \right)=\frac{\sigma (\lambda )}{\mu (\lambda )}$$

For a homogeneous object surface, the intrinsic reflectance spectrum should be approximately invariant across spatial locations; thus, residual spatial dispersion at each wavelength mainly reflects illumination- and geometry-induced inconsistencies. Consequently, a lower CV(λ) indicates more consistent spectra across positions and better correction performance. CV provides a normalized measure of dispersion and has been widely used to evaluate spectral stability and uniformity in hyperspectral and multispectral imaging systems [52,53]. Compared with absolute variance, CV is less sensitive to overall intensity scaling, making it well suited for comparing spectral consistency under varying illumination conditions. Lower CV values indicate higher spectral uniformity and, consequently, more effective spectral correction.

In addition to reporting the wavelength-wise CV curves, we summarize each experiment using an aggregated scalar score by summing the band-wise CV values over the full spectral range:

(13) $$Score=\sum_{\lambda }CV(\lambda )$$

This score captures the overall spectral variability accumulated across all wavelength bands, where lower values correspond to better spectral consistency.

**Figure 5:**
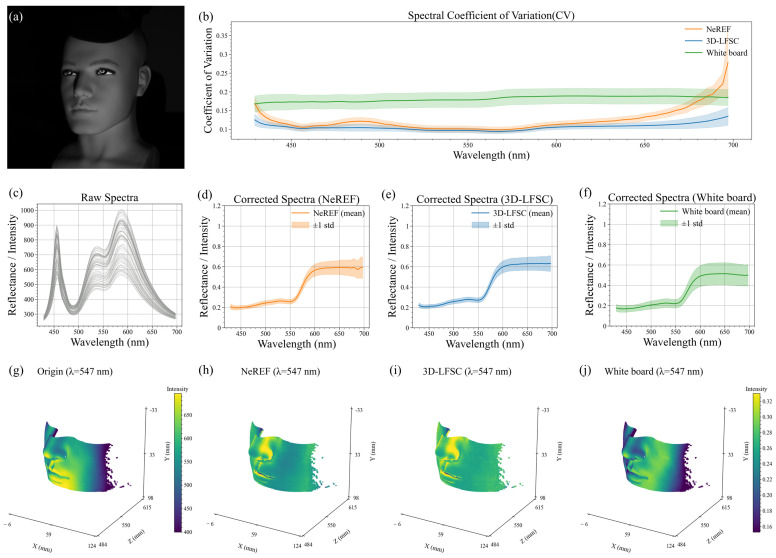
Spectral correction performance on the silicone face sample. (**a**) Image of the sample. (**b**) Wavelength-wise coefficient of variation (CV) for NeREF, 3D-LFSC, and white-board calibration, where lower values indicate better spectral consistency. (**c**) 100 camera-recorded raw spectra randomly sampled from different spatial locations. (**d**–**f**) Mean corrected spectra with ±1 standard deviation for NeREF, 3D-LFSC, and white-board calibration, respectively. (**g**–**j**) Spatial spectral-signal distributions at λ = 547 nm for the raw measurement and the three correction methods. The spatial maps are provided for qualitative visualization only and use the same colormap and an identical colorbar range. The primary quantitative comparison is based on the wavelength-wise CV curves and aggregated CV scores.

Figure 5a shows a photograph of the silicone face sample. We acquire 3D hyperspectral point clouds at multiple spatial position sets on the same sample to evaluate the correction performance under realistic surface geometry and reflectance-related properties.

The overall comparison is presented in Figure 5b, which plots the wavelength-wise CV curves with the corresponding variability band. 3D-LFSC consistently achieves lower CV values than NeREF across the entire spectral range, and its variability band is also generally narrower, indicating improved spectral consistency and robustness across wavelengths. In contrast, white-board calibration exhibits noticeably larger instability, particularly in terms of stronger wavelength-to-wavelength fluctuation.

To interpret these trends in terms of spectral shape, Figure 5c visualizes the distributions of camera-recorded raw spectral signals before correction, where substantial dispersion is evident among spectra sampled from different spatial positions. Figure 5d–f report the corrected spectral statistics (mean ± standard deviation) for the three methods. Compared with NeREF and white-board calibration, 3D-LFSC produces a tighter standard deviation envelope and more convergent corrected spectra, suggesting improved agreement among spectra from different spatial locations—consistent with the lower CV observed in Figure 5b.

Finally, we examine whether the methods can mitigate spatially non-uniform illumination. Figure 5g shows the spectral-signal map at 547 nm before correction, where the raw measurement exhibits pronounced bright and dark regions due to illumination non-uniformity and facial geometry. After applying NeREF (Figure 5h) and 3D-LFSC (Figure 5i), the spatial non-uniformity is reduced to some extent: most relatively flat regions are well corrected, while areas with abrupt geometric changes (e.g., the nose and lips) remain more challenging. In contrast, white-board calibration (Figure 5j) still shows evident signal non-uniformity. The spatial maps at 547 nm are provided as qualitative visualizations of the correction results. All spatial maps within the same figure were displayed using the same colormap and identical colorbar range. It should be noted that a single-wavelength spatial map alone is not sufficient to fully evaluate broadband spectral correction performance. Therefore, the main quantitative comparison is based on the wavelength-wise CV curves and the aggregated CV scores over the full spectral range. In regions with abrupt geometric variation, such as the nose and lips, both neural-network-based methods may still exhibit local overcompensation.

Table 3 reports the quantitative evaluation on the silicone face sample using the aggregated CV score defined in Equation (13). Each row corresponds to the same sample evaluated under a different spatial position set. For each set, we compute CV(λ) across the sampled spatial points at every wavelength and then sum the band-wise CV values over the full spectral range to obtain a single scalar score. Therefore, a lower score indicates better overall spectral consistency across spatial locations.

As shown in Table 3, 3D-LFSC yields lower scores than the NeREF baseline for all seven spatial position sets, demonstrating consistently improved spectral consistency under practical conditions. On average, the score decreases from 11.36 (NeREF) to 9.75 (3D-LFSC), corresponding to a 14.14% improvement relative to NeREF. Moreover, the variability across spatial position sets is reduced, with the standard deviation decreasing from 0.91 to 0.75 (17.43% reduction), indicating that 3D-LFSC not only improves the mean performance but also provides more stable results across different spatial configurations.

In contrast, white-board calibration performs substantially worse on this geometrically complex face sample. Its mean score increases to 16.68 (46.88% worse than NeREF), and the variability across spatial position sets rises markedly to 1.86 (105.28% worse than NeREF), suggesting limited robustness under geometry- and illumination-dependent effects. Overall, the numerical results in Table 3 corroborate the trends observed in Figure 5b, confirming that 3D-LFSC achieves both better average performance and improved stability on the silicone face sample.

In addition, a local-reference comparison was performed on four relatively flat or near-normal ROIs of the silicone face sample, where white-board calibration can serve as an approximate local reference. As shown in Supplementary Section S2 and Figure S1, the spectra corrected by 3D-LFSC are highly consistent with those obtained by white-board calibration in these regions, indicating that 3D-LFSC preserves local reference spectral characteristics while improving global spectral consistency over the entire complex surface.

**Figure 6:**
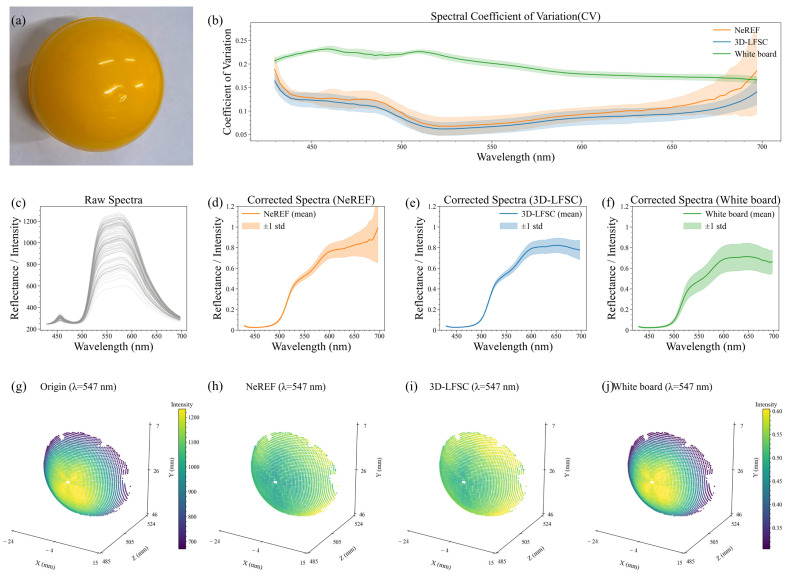
Spectral correction performance on the small-radius plastic sphere. (**a**) Image of the plastic sphere used in the experiment. (**b**) Wavelength-wise coefficient of variation (CV) for NeREF, 3D-LFSC, and white-board calibration, where lower values indicate better spectral consistency. (**c**) 100 camera-recorded raw spectra randomly sampled from different positions on the spherical surface. (**d**–**f**) Mean corrected spectra with ±1 standard deviation for NeREF, 3D-LFSC, and white-board calibration, respectively. (**g**–**j**) Spatial spectral-signal distributions at λ = 547 nm for the raw measurement and the three correction methods. The spatial maps are provided for qualitative visualization only and use the same colormap and identical colorbar range. The primary quantitative comparison is based on the wavelength-wise CV curves and aggregated CV scores.

For the small-radius plastic sphere (Figure 6), which represents an extreme curvature scenario, 3D-LFSC consistently achieves lower wavelength-wise CV values than NeREF, with a generally narrower variability band, indicating improved spectral consistency under severe geometric effects. The corrected spectra produced by 3D-LFSC also exhibit more compact mean ± standard deviation envelopes, reflecting better convergence across spatial position sets. For the small-radius plastic sphere, the spatial maps at 547 nm provide a qualitative visualization of the correction results. Both NeREF and 3D-LFSC substantially reduce the strong spatial non-uniformity observed in the raw measurement. However, because the spatial map represents only one selected wavelength, the correction performance is primarily evaluated using the wavelength-wise CV curves and the aggregated CV scores over the full spectral range. These quantitative results show that 3D-LFSC provides lower broadband spectral dispersion and better cross-position stability than the adapted NeREF baseline.

The quantitative results in Table 4 further confirm this trend. Compared with NeREF, 3D-LFSC reduces the mean aggregated score from 9.67 to 8.64, corresponding to a 10.74% improvement, and also decreases the variability across spatial position sets from 1.34 to 1.19 (10.72% reduction). In contrast, white-board calibration exhibits a much larger mean score (18.10, 87.10% worse than NeREF), indicating substantial degradation under strong curvature.

In summary, 3D-LFSC consistently improves spectral consistency compared with NeREF in both practical (silicone face) and extreme (small-radius sphere) scenarios. Specifically, it reduces the mean aggregated score (ΣCV) by 14.14% with a 17.43% reduction in cross-setting variability on the face sample, and by 10.74% with a 10.72% reduction on the sphere. These gains indicate that incorporating measurable 3D geometric information not only improves average correction quality but also enhances robustness to spatial position changes.

### 3.3. Color Detection via Corrected 3D Hyperspectral Point Clouds

To demonstrate the practical value of the corrected 3D hyperspectral point clouds, we further evaluated color consistency using hyperspectral measurements in the CIELAB color space. CIELAB color differences (ΔE) [54,55] for identical ROIs across spatial positions benchmark spectral stability, with methodology details in the Supplementary Materials. Because the adapted NeREF baseline did not provide sufficiently stable spectra for reliable CIELAB conversion under our 92-band setting, the color-consistency analysis in this section is reported only for 3D-LFSC and white-board calibration.

**Figure 7:**
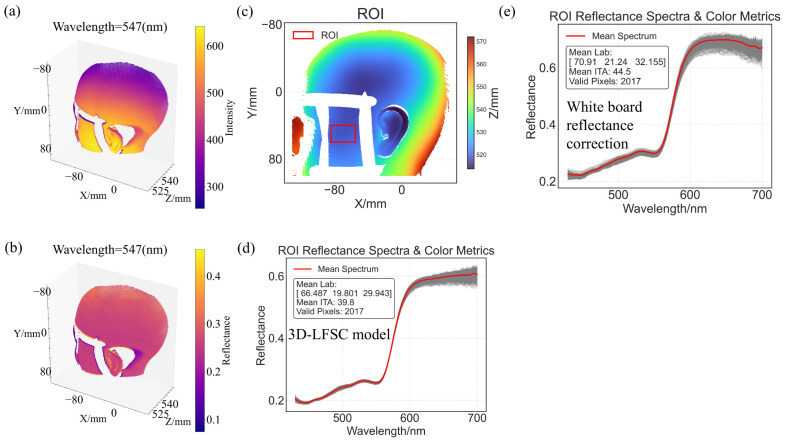
Illustrates the color-consistency validation results. (**a**,**b**) show the raw and corrected 3D hyperspectral point clouds at 547 nm for one representative position of the silicone face sample. The ROI is defined in (**c**). (**d**,**e**) show the CIELAB values of the ROI after spectral correction by the 3D-LFSC model and white-board calibration, respectively. The mean ΔE across five positions is reduced by 87% using 3D-LFSC compared with white-board calibration (Table 5), demonstrating improved color consistency under repeated spatial-position measurements.

Figure 7 illustrates the results of color detection. Figure 7a,b illustrate one of the positions of silicone face model’s 3D hyperspectral point clouds and corrected 3D hyperspectral point clouds at 547 nm. ROI is defined in Figure 7c. Figure 7d,e are the LAB values of ROI, whose spectra are processed by the 3D-LFSC model and white-board calibration respectively. Mean ΔE across five positions shows an 87% reduction with 3D-LFSC versus white-board calibration (Table 5), demonstrating significantly improved color consistency across different spatial positions.

**Figure 8:**
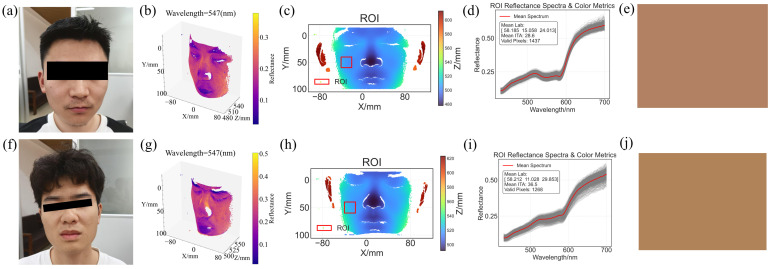
Color detection of human faces. (**a**–**e**) Results for Human Face 1: (**a**) RGB image; (**b**) spectrally corrected 3D hyperspectral point clouds (547 nm wavelength band); (**c**) Region of Interest (ROI); (**d**) mean spectrum and spectral distribution within the ROI; (**e**) detected color patch of the ROI. (**f**–**j**) Results for Human Face 2: (**f**) RGB image; (**g**) spectrally corrected 3D hyperspectral point clouds (547 nm wavelength band); (**h**) Region of Interest (ROI); (**i**) mean spectrum and spectral distribution within the ROI; (**j**) detected color patch of the ROI.

We also captured 3D hyperspectral point clouds of real human faces to evaluate the performance of the proposed framework, demonstrating its potential application in skin-related optical assessment. Figure 8 presents the color detection results. Two human faces (Figure 8a,f) were analyzed, with their 3D hyperspectral point clouds acquired in triplicate (Figure 8b,g). The corresponding color detection results for the Regions of Interest (ROI) are shown in Figure 8e,j. Identical ROIs were selected on both faces to calculate the color difference metric ΔE (Table 6). Our method enables quantitative calculation of facial color differences, demonstrating potential for skin index detection applications. All participants provided consent for publication.

## 4. Discussion and Conclusions

In summary, we propose a 3D hyperspectral measurement framework that produces high-depth-accuracy point clouds, high spectral resolution hyperspectral images, and spectrally corrected 3D hyperspectral point clouds. The framework consists of: (1) an optical system that jointly captures high-accuracy 3D geometry and high-resolution hyperspectral measurements while balancing system complexity and reconstruction accuracy; and (2) 3D-LFSC, a physics-guided model motivated by radiometric considerations of geometry-dependent reflection, which transforms camera-recorded spectral signals into geometry-normalized apparent reflectance estimates. Owing to the cross-polarized optical configuration, the obtained apparent reflectance estimates should be regarded as diffuse-dominant cross-polarized spectral quantities, rather than complete surface reflectance. This configuration is suitable for improving color and spectral consistency on complex surfaces, but it does not aim to recover the full angular reflectance behavior of the material.

Comprehensive validation demonstrates that the proposed framework achieves a mean sphere-fitting RMS residual of 36.39 μm and a spectral resolution of 7 nm. 3D-LFSC improves spectral consistency on geometrically complex surfaces by more than 10% compared with the adapted NeREF baseline. In particular, for spectral correction on complex surfaces, 3D-LFSC achieves over 10% improvement compared with NeREF; for color-consistency validation, 3D-LFSC reduces the mean ΔE by 87% compared with white-board calibration. These results highlight the potential of our framework for distinguishing objects that are difficult to differentiate visually. Overall, the proposed system enables the acquisition of high-quality 3D hyperspectral point clouds, providing a solid foundation for downstream hyperspectral analysis and processing.

Future system evolution will focus on three critical dimensions. In spectral detection, we plan to extend the operational wavelength range into the near-infrared region, enabling precise acquisition of spectral indicators such as tissue hemoglobin indices and vegetation water stress indices. This expansion will address the growing demand for broad-spectrum analysis in biomedical and agricultural remote sensing applications. For geometric precision, we propose to implement a graph neural network-based normal vector estimation algorithm resilient to surface discontinuities. This approach integrates local curvature constraints with global geometric continuity priors to effectively mitigate estimation inaccuracies in regions with abrupt surface variations. Regarding system architecture, we will advance to high-resolution snapshot hyperspectral imaging or compressive sensing hyperspectral cameras, overcoming the acquisition rate limitations of current line-scanning systems to achieve video-rate 3D hyperspectral point cloud capture at millisecond intervals. These synergistic advancements will collectively break new ground in spectral coverage, geometric accuracy, and temporal resolution, establishing a robust technical foundation for real-time precision monitoring of dynamic processes.

Moreover, to address the limitation of the current single-view system in validating multi-angle spectral accuracy, we plan to extend the system with a multi-view fusion capability in future work. Specifically, a high-precision motorized rotary stage will be integrated into the existing hardware platform. By sequentially acquiring 3D hyperspectral point clouds from multiple views and applying point-cloud registration and fusion algorithms, such as iterative closest point and global optimization, a complete multi-view 3D hyperspectral model can be constructed. This strategy will enable the spectral correction consistency of the 3D-LFSC model to be evaluated under different viewing angles, and may also improve reconstruction completeness and spectral measurement reliability for complex surfaces.

## Figures and Tables

**Table 1:** Performance of 3D hyperspectral measurement framework.

Specifications	Parameters
Effective spectral range	430–700 nm
Usable spectral channels	92
Spectral resolution	7 nm (Full Width at Half Maximum)
Sphere-fitting RMS residual	36.39 μm at 530 mm, mean of 9 measurements
Mean radius error	22.19 μm
Acquisition time	approximately 60 s
Working distance	400 mm~1000 mm

**Table 2:** System-level comparison of the proposed 3D hyperspectral measurement framework with representative 3D multispectral or hyperspectral imaging systems.

Work	Imaging Strategy	Spectral Performance	3D Accuracy	Method for Spectral Correction
Wan et al. [50]	Binocular multispectral imaging	20 nm, 10 channels	0.89 mm at 1036 mm	White-board calibration
Qi et al. [24]	4D multi-frame co-encoded spectral imaging	420–660 nm; average SAM = 4.28	0.1 mm	Not specifically considered
Gevaux et al. [51]	3D facial hyperspectral imaging with LCTF	10 nm, 31 channels	~0.4 mm	Geometrical calibration; limited performance on some facial regions
Luo et al. [27]	Portable 4D snapshot hyperspectral imaging	10 nm	55.7 μm	White-board calibration
**This work**	Structured-light 3D & HSI fusion	**7 nm, 92 channels**	**36.39 μm sphere-fitting RMS residual at 530 mm**	**3D-LFSC geometry-aware correction**

**Table 3:** Performance comparison via silicone face sample. The scores are computed through Equation (13).

Spatial Position Set	Score (NeREF)	Score (3D-LFSC)	Score (White-Board)
1	11.07	8.81	18.05
2	11.76	9.71	16.86
3	11.65	11.06	14.29
4	10.87	9.59	17.39
5	13.08	10.52	19.09
6	11.19	9.65	17.50
7	9.89	8.93	13.59
Mean Score	11.36	9.75	16.68
σ(Score)	0.91	0.75	1.86
Mean Score vs. NeREF	0.00%	14.14%	−46.88%
σ(Score) vs. NeREF	0.00%	17.43%	−105.28%

**Table 4:** Performance comparison via plastic sphere.

Spatial Position Set	Score (NeREF)	Score (3D-LFSC)	Score (White-Board)
1	10.22	7.78	17.79
2	8.84	7.01	18.86
3	12.06	10.52	18.63
4	8.68	8.84	17.78
5	8.57	9.02	17.45
Mean Score	9.67	8.64	18.10
σ(Score)	1.34	1.19	0.55
Mean Score vs. NeREF	0.00%	10.74%	−87.10%
σ(Score) vs. NeREF	0.00%	10.72%	59.13%

**Table 5:** CIELAB values of silicone face model’s ROI at 5 positions.

Sample	3D-LFSC Model				White-Board Calibration			
	L*	a*	b*	∆E	L*	a*	b*	∆E
1	66.498	19.935	30.013	0.149	70.975	21.381	32.232	2.779
2	66.916	19.998	30.126	0.340	70.392	21.144	31.800	2.038
3	66.924	20.263	30.479	0.594	71.539	21.517	32.027	3.250
4	66.414	20.084	29.809	0.290	64.161	19.654	29.359	4.818
5	66.208	19.962	29.751	0.486	65.585	19.970	29.979	3.236

**Table 6:** CIELAB values of human faces’ ROI.

Sample	Human Face 1				Human Face 2				∆E Between Face 1 and Face 2
	L*	a*	b*	∆E	L*	a*	b*	∆E	
1	58.185	15.058	24.013	0.290	58.317	11.541	30.313	0.137	7.068
2	58.569	14.873	24.036	0.166	58.429	11.314	30.211	0.163	6.753
3	58.514	14.82	24.252	0.202	58.442	11.565	30.148	0.128	6.771

## Data Availability

The data and code supporting the findings of this study are available at Figshare: https://doi.org/10.6084/m9.figshare.30995257 (accessed on 19 May 2026).

## Supplementary Materials

The following supporting information can be downloaded at: https://www.mdpi.com/article/10.3390/s26113396/s1, Figure S1: Local-reference comparison between 3D-LFSC and white-board calibration on the silicone face sample; Table S1: Calibration results of 3D hyperspectral measurement framework; Table S2: Repeated measurements of the certified ceramic sphere. The reference radius of the certified ceramic sphere was 15.00375 ± 0.0025 mm. The radius error was calculated as Equation (S1).
