# Supplementary material for: Close-Range 3D Hyperspectral Measurement System with a Physics-Guided Spectral Correction Model
Source: Sensors (Basel). 2026 May 27;26(11):3396. doi: 10.3390/s26113396 (PMC13259459; doi:10.3390/s26113396)
Supplement: Supplementary file 1 [file sensors-26-03396-s001.zip › sensors-4297284-supplementary.pdf]

# Supplementary Material

## 1 System parameters and calibration results

### *1.1 System calibration parameters*

Table S1. Calibration results of 3D hyperspectral measurement framework

| System parameters | Result |
|---|---|
| Camera distortion | $D_c = [-0.069083 \quad 0.629503 \quad 0.000972 \quad 0.000491 \quad -2.523322]$ |
| Camera Intrinsic parameters | $A_C = \begin{bmatrix} 3513.836841 & 0 & 1211.808534 \\ 0 & 3512.100550 & 1063.184630 \\ 0 & 0 & 1 \end{bmatrix}$ |
| Hyperspectral camera distortion | $D_h = [-0.344425 \quad 2.300607 \quad 0.002323 \quad -0.001202 \quad -15.185296]$ |
| Hyperspectral camera Intrinsic parameters | $A_H = \begin{bmatrix} 975.773138 & 0 & 251.921779 \\ 0 & 982.359736 & 259.6254320 \\ 0 & 0 & 1 \end{bmatrix}$ |
| Projector Intrinsic parameters | $A_P = \begin{bmatrix} 1557.104016 & 0 & 1010.647101 \\ 0 & 982.359736 & 259.625432 \\ 0 & 0 & 1 \end{bmatrix}$ |
| Relative rotation matrix between hyperspectral camera and camera | $R_{HC} = \begin{bmatrix} 0.999802 & -0.010822 & 0.016715 \\ 0.011250 & 0.999606 & -0.025724 \\ -0.016430 & 0.025907 & 0.999529 \end{bmatrix}$ |
| Relative translation vector between hyperspectral camera and camera | $T_{HC} = [-12.936616 \quad -0.982575 \quad -1.034244]^T$ |
| Relative rotation matrix between projector and camera | $R_{PC} = \begin{bmatrix} -0.999827 & 0.009098 & -0.016221 \\ -0.005895 & -0.982232 & -0.187578 \\ -0.017639 & -0.187450 & 0.982116 \end{bmatrix}$ |
| Relative translation vector between projector and camera | $T_{PC} = [-2.951731 \quad 198.879110 \quad -59.987626]^T$ |

### *1.2 Repeated measurement of the certified ceramic sphere*

To further evaluate the 3D reconstruction performance of the structured-light module, repeated measurements were conducted using a certified ceramic sphere with a diameter of $30.0075 \pm 0.005$ mm, corresponding to a reference radius of $15.00375 \pm 0.0025$mm. The sphere was placed on the same measurement plane but at different lateral positions within the system field of view. This measurement arrangement was used to evaluate the stability of the reconstructed geometry across different spatial positions under the same working distance.

For each measurement, the reconstructed point cloud of the ceramic sphere was imported into CloudCompare software, where a best-fit sphere was fitted to the measured 3D points. The fitted radius and the sphere-fitting root-mean-square (RMS) residual were then recorded. The sphere-fitting RMS residual represents the point-to-sphere residual dispersion and was used to characterize the local surface reconstruction precision and depth repeatability. In addition, the radius error was calculated as the absolute difference between the fitted radius and the reference radius:

$$E_r = | r_{\text{fit}} - r_{\text{ref}} | \quad \text{(S1)}$$

where $r_{\mathrm{fit}}$ is the fitted radius obtained from CloudCompare, and $r_{\mathrm{ref}}$ is the certified reference radius of the ceramic sphere. Compared with the RMS residual, the radius error provides a complementary indicator of dimensional accuracy. The results of nine repeated measurements are summarized in Table S2.

Table S2. Repeated measurements of the certified ceramic sphere. The reference radius of the certified ceramic sphere was 15.00375 ± 0.0025mm. The radius error was calculated as Eq (S1).

| Trial | Fitted radius (mm) | Sphere-fitting RMS residual (μm) | Radius error (μm) |
|---|---|---|---|
| 1 | 15.01180 | 33.03 | 8.05 |
| 2 | 14.97780 | 35.73 | 25.95 |
| 3 | 14.97170 | 36.35 | 32.05 |
| 4 | 14.96480 | 31.47 | 38.95 |
| 5 | 15.03200 | 42.60 | 28.25 |
| 6 | 15.03210 | 40.58 | 28.35 |
| 7 | 14.99740 | 36.93 | 6.35 |
| 8 | 14.97700 | 27.50 | 26.75 |
| 9 | 14.99870 | 43.35 | 5.05 |
| **Mean** | **14.99592** | **36.39** | **22.19** |
| **σ** | **0.023838** | **4.94** | **11.70** |

## 2 Comparison with white-board calibration in near-normal regions

To further evaluate whether the proposed 3D-LFSC model preserves the local spectral characteristics in regions where conventional white-board calibration can provide an approximate reference, an additional local-reference comparison was performed on the silicone face sample. As shown in Figure S1a, four regions of interest (ROIs) were selected from relatively flat or near-normal areas of the silicone face point cloud. These regions were chosen because the effects of surface tilt and geometric shading are relatively weak, and therefore white-board calibration can be regarded as an approximate local reference.

For each ROI, nine neighboring points around the selected position were used to calculate the mean corrected spectrum. The spectra obtained by 3D-LFSC were then compared with those obtained by white-board calibration. To quantify the difference between the two methods, the root-mean-square error (RMSE) and spectral angle mapper (SAM) were calculated as:

$$RMSE = \sqrt{\frac{1}{N}\sum_{i=1}^{N}[R_{3D-LFSC}(\lambda_i) - R_{WB}(\lambda_i)]^2} \quad \text{(S2)}$$

$$SAM = cos^{-1}\left[\frac{\sum_{i=1}^{N} R_{3D-LFSC}(\lambda_i)R_{WB}(\lambda_i)}{\sqrt{\sum_{i=1}^{N} R_{3D-LFSC}^2(\lambda_i)}\sqrt{\sum_{i=1}^{N} R_{WB}^2(\lambda_i)}}\right] \quad \text{(S3)}$$

where $R_{\mathrm{3D\text{-}LFSC}}(\lambda_i)$ and $R_{\mathrm{WB}}(\lambda_i)$ denote the spectra corrected by 3D-LFSC and white-board calibration at wavelength $\lambda_i$, respectively, and $N$ is the number of spectral bands.

As shown in Figure S1b, the corrected spectra obtained by 3D-LFSC are highly consistent with those obtained by white-board calibration in the selected near-normal regions. The small RMSE values indicate that the amplitude differences between the two methods are limited, while the low SAM values indicate that the spectral shapes are well preserved. These results suggest that 3D-LFSC does not introduce obvious spectral distortion in local regions where white-board calibration is reliable. Instead, it preserves the local reference

spectral characteristics while providing improved spatial spectral consistency over the entire complex facial surface.

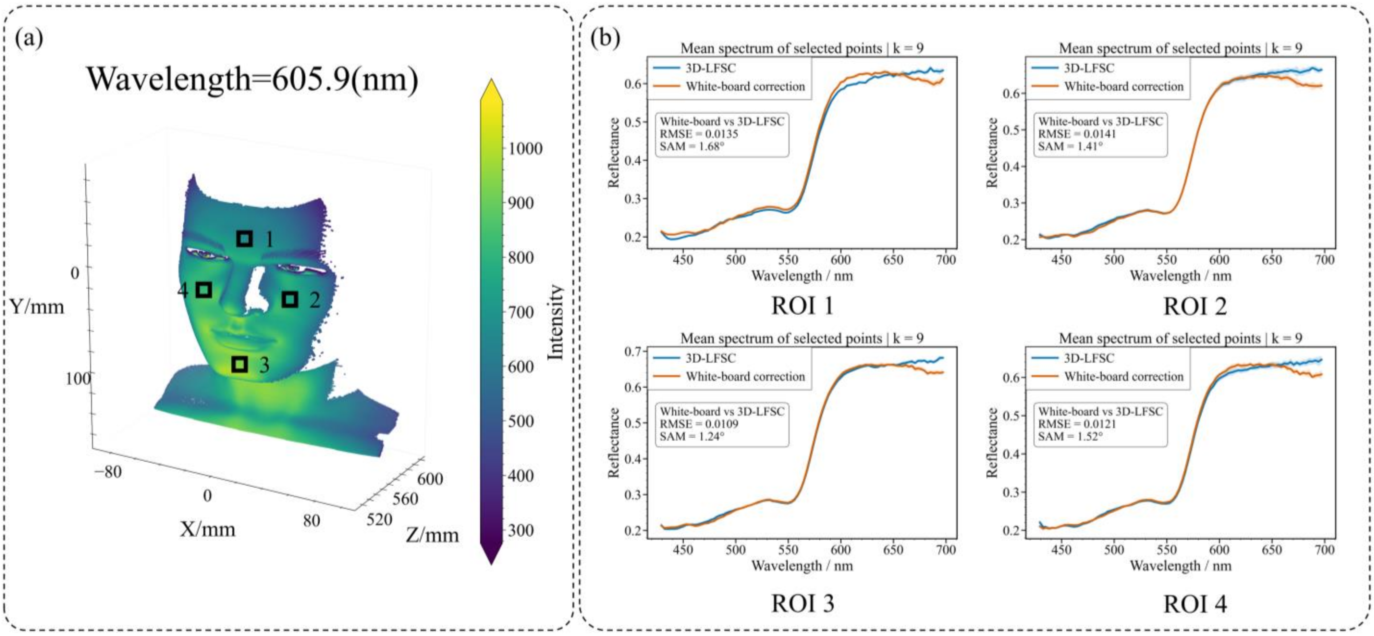


Figure S1. Local-reference comparison between 3D-LFSC and white-board calibration on the silicone face sample. (a) Four selected regions of interest (ROIs) on the silicone face point cloud at 605.9 nm. The selected ROIs correspond to relatively flat or near-normal local regions, where white-board calibration can serve as an approximate local reference. (b) Mean spectra of nine neighboring points in ROI 1–ROI 4, respectively, after correction by 3D-LFSC and white-board calibration. The root-mean-square error (RMSE) and spectral angle mapper (SAM) between the two corrected spectra were calculated for each ROI. The close agreement between the two methods indicates that 3D-LFSC preserves the local reference spectral shape in near-normal regions while improving spectral consistency over complex facial geometry.
